# Falls Risk Assessment Compliance and Outcomes in Psychiatry of Old Age Inpatient Department

**DOI:** 10.1192/bjo.2026.11678

**Published:** 2026-06-30

**Authors:** Huda Khan, Aine Butler

**Affiliations:** 1 National Forensic Mental Health Service, Dublin, Ireland; 2 North Dublin Mental Health Services for Older Persons, Dublin, Ireland

## Abstract

**Aims:**

The primary aim of this audit is to determine the extent to which falls risk assessments are completed within 24 hours of admission for patients in the Psychiatry of Old Age inpatient department, and to assess whether identified risks lead to timely and appropriate interventions. According to the Standards based on NICE guidelines (CG161)

**Methods:**

All patients aged 65 years and older admitted to the 8-bed Psychiatry of Old Age inpatient unit during the audit period were included in this audit. Data was collected retrospectively using a standardized audit tool through review of admission notes, falls risk assessment documentation, multidisciplinary care plans, and incident reports. The audit tool captured patient demographics, date of admission, timing of completion of falls risk assessments, identified individual risk factors, and falls prevention interventions implemented during the inpatient stay. In addition, the number and timing of any falls occurring during admission was recorded to evaluate the relationship between risk identification, intervention implementation, and fall incidents. Patients transferred from another inpatient facility more than 48 hours after admission, where a falls risk assessment had already been completed prior to transfer, was excluded to ensure consistency in baseline assessment and care processes.

**Results:**

Falls risk assessments were completed for 5 patients. In 4 patients (80%), the assessment was conducted within 24 hours of admission, and identified risks were addressed through documented interventions within 48 hours. Clinical documentation, including progress notes, was present for all patients (100%).

A total of 4 falls were recorded during the admission period across all patients. All falls were appropriately documented in the clinical records (100%).

**Conclusion:**

Overall, Cycle One findings were compliant with the agreed standards.However, education was provided to team members to reinforce the importance of timely and accurate falls risk assessment. Areas for improvement were also identified in relation to the quality and consistency of documentation. These points will be addressed and reviewed in the next audit cycle. Date collection of cycle 2 is under process.

